# The impact of disease severity on paradoxical association between body mass index and mortality in patients with acute kidney injury undergoing continuous renal replacement therapy

**DOI:** 10.1186/s12882-018-0833-5

**Published:** 2018-02-07

**Authors:** Hyoungnae Kim, Hyunwook Kim, Misol Lee, Min-Uk Cha, Ki Heon Nam, Seong Yeong An, Su-Young Jung, Jong Hyun Jhee, Seohyun Park, Hae-Ryong Yun, Youn Kyung Kee, Hyung Jung Oh, Jung Tak Park, Tae Ik Chang, Tae-Hyun Yoo, Shin-Wook Kang, Seung Hyeok Han

**Affiliations:** 1 Department of Internal Medicine, Institute of Kidney Disease Research, Yonsei University College of Medicine, 50-1 Yonsei-ro, Seodaemun-Gu, Seoul, 03722 South Korea; 2 Division of Nephrology, Soonchunhyang University, Seoul, Republic of Korea; 3 Ewha Institute of Convergence Medicine, Ewha Womans University Mokdong Hospital, Seoul, Republic of Korea; 4 Division of Nephrology, Department of Internal Medicine, National Health Insurance Service Medical Center, Ilsan Hospital, Goyang, Gyeonggi-do Republic of Korea

**Keywords:** Acute kidney injury, Body mass index, Continuous renal replacement therapy, Disease severity, Mortality

## Abstract

**Background:**

Association between high body mass index (BMI) and survival benefit is confounded by comorbid conditions such as nutritional status and inflammation. Patients with acute kidney injury (AKI), particularly those receiving continuous renal replacement therapy (CRRT), are highly catabolic and more susceptible to loss of energy. Herein, we evaluated whether disease severity can modify the relationship between BMI and mortality.

**Methods:**

We conducted an observational study in 1144 patients who had undergone CRRT owing to various causes of AKI between 2010 and 2014. Patients were categorized into four groups; underweight (< 18.5 kg/m^2^), normal (18.5–22.99 kg/m^2^), overweight (23.0–24.99 kg/m^2^), and obesity (≥25 kg/m^2^) according to BMI classification by the Committee of Clinical Practice Guidelines and Korean Society for the Study of Obesity. More severe disease was defined as sepsis-related organ failure assessment (SOFA) score of ≥ a median value of 12. The study endpoint was death that occurred within 30 days after the initiation of CRRT.

**Results:**

The mean age was 63.2 years and 439 (38.4%) were females. The median BMI was 23.6 (20.9–26.2) kg/m^2^. The obese group were younger and higher SOFA score than normal BMI group. In a multivariable Cox regression analysis, we found a significant interaction between BMI and SOFA score (*P* <  0.001). Furthermore, obese patients were significantly associated with a lower risk of death as compared to normal BMI group after adjusting confounding factors [hazard ratio (HR), 0.81; 95% confidence interval (CI), 0.68–0.97; *P* = 0.03]. This association was only evident among patients with high severity (HR, 0.61; 95% CI, 0.48–0.76, *P* <  0.001). In contrast, in those with low severity, survival benefit of high BMI was lost, whereas underweight was associated with an increased risk of death (HR, 1.74; 95% CI, 1.16–2.60; *P* = 0.007).

**Conclusion:**

In this study, we found a survival benefit of high BMI in AKI patients undergoing CRRT, particularly in those with more disease severity; the effect was not observed in those with less disease severity.

**Electronic supplementary material:**

The online version of this article (10.1186/s12882-018-0833-5) contains supplementary material, which is available to authorized users.

## Background

Obesity has recently emerged as an important public health problem worldwide. It is indeed associated with hypertension, dyslipidaemia, and diabetes mellitus (DM) and obese individuals who have these comorbid conditions are at a high risk of developing cardiovascular and cerebrovascular diseases [1]. Obesity is also highly associated with the development of chronic kidney disease (CKD), microalbuminuria, and overt proteinuria [2]. Interestingly, obesity is commonly observed in many critically ill patients who are admitted to the intensive care unit (ICU). In a previous meta-analysis from the United States, approximately 30% of ICU patients had a body mass index (BMI) of ≥30 kg/m^2^, and duration of mechanical ventilation and length of ICU stay were longer in these patients [3].

Acute kidney injury (AKI) commonly occurs and is a serious problem especially in critically ill patients because complications caused by AKI can lead to adverse outcomes such as increased length of hospital stay, high mortality, and progression to CKD [4]. Because obese patients are burdened with high comorbidities, they are more likely to develop AKI and suffer from more serious complications than non-obese patients. However, there has been conflicting results on the association between AKI, obesity, and mortality. In fact, previous studies have shown that obese patients had high incidence of AKI and more severe kidney injury [5–8], resulting in increased mortality [6, 9, 10]. Nevertheless, there have been several reports that obesity exhibited an inverse or null relationship with mortality [5, 11–15]. In fact, some recent studies clearly showed that critically ill patients with obesity have survival benefit while they are treated in the ICU [7, 16].

One possible mechanism of obesity paradox is that high nutritional reserve of obese patients plays an important role during acute life-threatening illness [17]. Interestingly, recent observational studies reported that the relationship between obesity and mortality is confounded by comorbid conditions. In these studies, survival benefit of obese patients was observed only in patients with high C-reactive protein (CRP), but not in those with low CRP [18]. In addition, among patients with obesity, malnourished patients had worse outcomes than those in relatively good nutritional status [19]. Of note, patients with AKI are more likely to have high comorbidities and high disease severity. In particular, these patients are highly inflamed and deprived of nutrition because of increased protein catabolism and high energy consumption. This process can be more deteriorated when disease severity is high. Thus, we hypothesized that survival benefit of obesity is more evident under high disease severity condition. In this study, we sought to examine whether disease severity can modify the relationship between obesity and mortality in critically ill patients and conducted an observational study in patients with AKI who underwent continuous renal replacement therapy (CRRT).

## Methods

### Patient selection

**Fig. 1:**
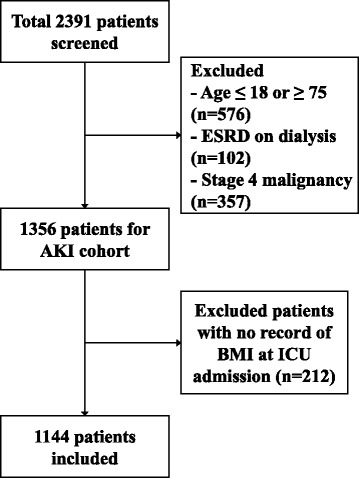
Flowchart of participants in the cohort. *Abbreviations*: AKI, acute kidney injury; ESRD, end stage renal disease; BMI, body mass index

This study retrospectively examined the relationship between body mass index and mortality by disease severity. Figure 1 presents a flow chart depicting the selection of subjects. We selected 1144 adult patients who were treated with CRRT in the ICU at two medical centers, Yonsei University Severance Hospital and National Health Insurance Service Medical Center Ilsan hospital between January 2010 and December 2014. Among 2391 patients who were initially assessed for study eligibility, 1247 patients who met following criteria were excluded: 1) ≤18 or ≥75 years old, 2) end-stage renal disease (ESRD) on dialysis, 3) stage 4 malignancy, or 4) no data for BMI. The study was approved by the Institutional Review Board (IRB) of each center. Since current study was a retrospective observational study and the study subjected de-identified, the IRB waived the need for written consent from the patients.

### Data collection

We reviewed electronic medical records and collected demographic and laboratory data. BMI was determined based on the formula of weight (kg)/height (m^2^). Weight and height of all patients were measured upon ICU admission. To evaluate the severity of the patients’ comorbidities, the Charlson comorbidity index (CCI) was calculated by using the score system as previously proposed [20]. Blood tests were done immediately after ICU admission and used for baseline data. The measured laboratory data included white blood cell (WBC) count, haemoglobin, haematocrit, platelet, prothrombin time, partial thromboplastin time, CRP, cholesterol, albumin, blood urea nitrogen, and creatinine. The Modification of Diet in Renal Disease equation [21] was used to determine the baseline estimated glomerular filtration rate (eGFR). The sepsis-related organ failure assessment (SOFA) score and Acute Physiology and Chronic Health Evaluation (APACHE) II score were also calculated based on data for the average vital signs, PaO2, FiO2, and Glasgow coma scale score in the first 24 h of ICU admission.

### CRRT protocol

Upon the development of AKI in ICU patients, nephrologists intervened and decided whether or not to begin CRRT in those who were critically ill. Generally, CRRT was applied in patients with sustained oliguria, uncontrolled volume overload, hyperkalemia or metabolic acidosis. All patients received continuous veno-venous haemodiafiltration by using a Prisma (Gambro Co., Ltd., Hechingen, Germany), Prismaflex (Gambro Co., Ltd), or multiFiltrate (Fresenius Medical Care GmbH, Bad Homburg, Germany) machine, through the internal jugular, subclavian, or femoral vein. CRRT machines were installed, and the system was maintained by trained and educated nurses. Biocompatible polyethersulphone membranes were used in all CRRTs. Pre-filter replacement was loaded with bicarbonate-containing fluid, and a pre-dilution method was applied. CRRT was started at a blood flow rate of 100 mL/min, and this was increased up to 150 mL/min. The total effluent volume as a sum of dialysis and replacement dose was targeted to deliver ≥35 mL⋅kg^− 1^⋅h^− 1^ in all patients.

### Definition

According to the World Health Organization (WHO) BMI classification, the criteria for obesity and overweight are ≥30 kg/m^2^ and ≥25 kg/m^2^, respectively. However, these cut-off values can be lowered for Asian populations. The prevalence of obesity in Asians by the conventional criteria is much lower and Asians confront higher metabolic risk even under BMI of 30 kg/m^2^ compare to other ethnic groups [22]. In fact, Korean individuals with a BMI of 23 to 24.9 kg/m^2^ are at higher risk of type 2 diabetes and hypertension as compared to those within the normal BMI range. Therefore in 2014, the Committee of Clinical Practice Guidelines and Korean Society for the Study of Obesity [23] proposed BMI classification as follows; underweight (< 18.5 kg/m^2^), normal (18.5–22.99 kg/m^2^), overweight (23.0–24.99 kg/m^2^), and obesity (≥25 kg/m^2^). In this study, we followed these criteria to define obesity. In addition, disease severity was assessed by using SOFA score. More severe disease was defined as SOFA score ≥ a median value of 12.

### Primary outcome

The primary outcome was overall survival measured from the date of the initiation of CRRT until death or day 30.

### Statistical analyses

Continuous variables were expressed as mean ± SD, and compared with *t*-test and one-way ANOVA. To compare between subgroups, post-hoc analysis was performed with Bonferroni analysis. The normality of the distribution of parameters was analysed by using the Kolmogorov-Smirnov test. If data did not show a normal distribution, these were presented as median and interquartile range and compared by using the Mann–Whitney test or Kruskal–Wallis test. Categorical variables were expressed as percentages and compared with the chi-square test.

To evaluate the relationship of BMI with covariables, and mortality, multivariable-adjusted Cox-proportional hazard models were constructed, and the results were presented as a hazard ratio (HR) and 95% confidence interval (CI). In model 1, we entered age, sex, CCI score, septic AKI, mean arterial pressure (MAP), eGFR, and SOFA score. We additionally adjusted WBC and albumin as an inflammatory marker and a nutritional marker, respectively, in model 2, and CRRT prescription was further added in model 3. To evaluate the impact of disease severity on the relationship between BMI and mortality, we first examined interaction between BMI and SOFA score in Cox proportional hazard models. Then, the same analyses were performed according to disease severity status and presence of comorbidities.

In sensitivity analyses, we re-categorized patients by another BMI criteria. The WHO recommends that additional cut-off points of obesity such as 23, 27.5, 32.5 and 37.5 kg/m^2^ can be used for public health action [24]. Thus, we applied these criteria to confirm our findings. Furthermore, we re-defined disease severity according to APACHE II score. The high disease severity was defined as APACHE II score of ≥ a median value of 28, and Cox regression analyses were repeated after switching SOFA score to APACHE II score. Finally, we performed restricted cubic splines to examine whether the association between disease severity and mortality may differ depending on the presence of obesity. Statistical significance was defined as *P* <  0.05. All analyses were conducted by using SPSS, version 23.0 (IBM Corporation, Armonk, NY, USA), SAS version 9.4 (SAS Institute, Cary, NC, USA), and GraphPad Prism version 5.0 (GraphPad Software Inc., San Diego, CA, USA).

## Results

### Patient characteristics

The baseline characteristics of the patients according to BMI are presented in Table 1. The mean age was 63.2 years, and 439 patients (38.4%) were women. The obese group was younger than normal (*P* = 0.02) and overweight group (*P* = 0.02). The average BMI was 23.6 (20.9–26.3) kg/m^2^. The mean CCI score was 3.2 ± 2.2 and was similar between groups. Sepsis was a predominant cause of ICU admission and 797 patients (69.7%) had undergone sepsis. The most common cause of CRRT was sustained oliguria and did not different between groups. The dose of CRRT was 43.4 ± 16.9 mL/kg and obese group received significantly lower CRRT dose than underweight (*P* = 0.006) and normal group (*P* <  0.001). The average SOFA and APACHE II score were 12.1 ± 3.6 and 27.1 ± 8.4, respectively. The SOFA score was significantly higher in obese group than in underweight (*P* = 0.02) and normal group (*P* = 0.03); however, APACHE II score did not differ between groups (*P* = 0.44). The baseline characteristics according to disease severity are presented in Table 2. BMI was significantly higher in the high severity group than in the low severity group [23.4 (20.8–25.9) vs. 23.9 (21.2–26.7) kg/m^2^, *P* = 0.009]. The patients in the low disease severity group had higher prevalence of DM and cardiovascular disease, whereas cancer was more prevalent in the high severity group. As a result, the low severity group had a significantly lower CCI score than the higher severity group (*P* = 0.002). In addition, eGFR and the level of total cholesterol and hemoglobin were lower in the high severity group than in the low severity group. As expected, percentages of patients treated with vasopressor and mechanical ventilation were significantly higher in the high severity group than in the lower severity group (*P* <  0.001, respectively).

**Table 1:** Baseline characteristics of patients according to BMI classification

	BMI classification				Total	*p*-value
	Underweight	Normal	Overweight	Obesity		
Number	99	403	220	422	1144	
BMI (kg/m^2^)^a^	16.9 (15–17.8)	21.1 (20.1–22.2)	24.1 (13.5–24.5)	27.5 (25.9–29.2)	23.6 (20.9–26.3)	< 0.001
Age (yr)	62.3 ± 17.2	65.0 ± 13.6	63.9 ± 14.0	61.3 ± 14.5	63.2 ± 14.4	0.002
Sex (Female, %)	38 (38.4)	162 (40.2)	65 (29.5)	174 (41.3)	439 (38.4)	0.03
DM (*n*, %)	27 (27.6)	140 (34.7)	79 (35.9)	153 (36.3)	399 (34.9)	0.42
HTN (*n*, %)	40 (40.4)	217 (53.8)	110 (50.0)	233 (55.3)	600 (52.5)	0.05
MI (*n*, %)	10 (10.1)	47 (11.7)	17 (7.7)	37 (8.8)	111 (9.7)	0.37
CHF (*n*, %)	20 (20.2)	71 (17.6)	37 (16.8)	57 (13.5)	185 (16.2)	0.26
CVA (*n*, %)	7 (7.1)	50 (12.4)	25 (11.4)	35 (8.3)	117 (10.2)	0.08
PVD (*n*, %)	6 (6.1)	15 (3.7)	12 (5.5)	13 (3.1)	46 (4.0)	0.35
COPD (*n*, %)	11 (11.0)	33 (8.2)	13 (5.9)	23 (5.5)	80 (7.0)	0.15
Cancer (*n*, %)						
Solid tumor	18 (18.4)	96 (23.8)	51 (23.3)	93 (22.1)	258 (22.6)	
Metastasis	5 (5.1)	19 (4.7)	18 (8.2)	26 (6.2)	68 (6.0)	0.77
Leukemia	3 (3.1)	19 (4.7)	13 (5.9)	16 (3.8)	51 (4.5)	
Lymphoma	12 (12.2)	37 (9.2)	21 (9.6)	38 (9.0)	108 (9.5)	
CCI score	3.1 ± 2.4	3.2 ± 2.2	3.1 ± 2.2	3.2 ± 2.3	3.2 ± 2.2	0.94
Sepsis (*n*, %)	74 (74.7)	294 (73.0)	149 (67.7)	280 (66.5)	797 (69.7%)	0.13
Postop AKI (*n*, %)	3 (3.0)	13 (3.2)	9 (4.1)	26 (6.2)	51 (4.5)	0.18
Cause of CRRT						
Volume overload	8 (8.1)	62 (15.3)	26 (11.8)	64 (15.2)	160 (13.9)	
Metabolic acidosis	26 (26.3)	85 (21.1)	43 (19.5)	88 (20.8)	242 (21.2)	
Hyperkalemia	2 (2.0)	19 (4.7)	12 (5.4)	25 (5.9)	58 (5.1)	0.86
Uremia	13 (13.1)	35 (8.7)	21 (9.5)	46 (10.9)	115 (10.1)	
Oliguria	35 (35.4)	112 (27.5)	54 (24.5)	93 (22.0)	294 (25.7)	
Others	15 (15.2)	90 (22.4)	64 (29.1)	106 (25.1)	275 (24.0)	
CRRT dose (mL/kg)	46.2 ± 16.4	44.6 ± 19.4	41.3 ± 15.1	40.0 ± 15.0	43.4 ± 16.9	< 0.001
MAP (mmHg)	76.6 ± 17.5	77.4 ± 14.2	77.3 ± 14.3	77.7 ± 14.5	77.5 ± 14.6	0.92
Creatinine (mg/dL)	2.6 ± 1.5	2.5 ± 1.3	2.9 ± 1.9	2.9 ± 1.7	2.7 ± 1.6	0.001
eGFR (mL/min/1.73m^2^)	33.2 ± 18.0	33.1 ± 22.3	32.7 ± 24.4	29.0 ± 18.8	31.5 ± 21.4	0.03
Albumin (g/dL)	2.5 ± 0.5	2.6 ± 0.6	2.6 ± 0.6	2.6 ± 0.6	2.6 ± 0.6	0.47
Total cholesterol (mg/dL)	111.9 ± 55.9	107.6 ± 48.5	98.1 ± 46.5	108.1 ± 68.9	106.2 ± 57.3	0.56
WBC (× 10^3^/mm^3^)	15.3 ± 25.9	13.8 ± 11.7	14.7 ± 12.8	13.7 ± 9.9	14.1 ± 13.2	0.64
Hemoglobin (g/dL)	9.7 ± 2.4	9.7 ± 2.1	9.4 ± 2.4	9.6 ± 2.3	9.6 ± 2.3	0.56
CRP (mg/L)^a^	37.1 (10.6–213.8)	87.3 (17.7–171.7)	72.9 (18.6–255.7)	56.7 (14.2–151.8)	67.5 (15.4–164.1)	0.06
Vasopressor (*n*, %)	68 (68.9)	270 (67.0)	165 (75.3)	314 (74.4)	817 (71.4)	0.15
Ventilator (*n*, %)	80 (81.0)	345 (85.7)	184 (83.7)	352 (83.5)	961 (84.0)	0.74
SOFA	11.3 ± 3.4	11.8 ± 3.7	12.0 ± 3.5	12.5 ± 3.5	12.1 ± 3.6	0.005
APACHE II	26.4 ± 8.3	27.4 ± 8.0	27.5 ± 9.1	26.7 ± 8.4	27.1 ± 8.4	0.44

*Abbreviations*: *DM* diabetes mellitus, *HTN* hypertension, *MI* myocardiac infarction, *CHF* congestive heart failure, *CVA* cerebrovascular attack, *PVD* peripheral vascular disease, *COPD* chronic obstructive pulmonary disease, *CCI* Charlson comorbidity index, *AKI* acute kidney injury, *BMI* body mass index, *MAP* mean arterial pressure, *eGFR* estimated glomerular filtration rate, *WBC* white blood cell, *SOFA* sepsis-related organ failure assessment

^a^Data were expressed as a median and interquartile range and compared by Kruskal-Wallis test

**Table 2:** Baseline characteristics of patients according to disease severity groups

	Disease severity		*p*-value
	Low severity (SOFA < 12)	High severity (SOFA ≥12)	
Number	601	543	
BMI (kg/m^2^)^a^	23.4 (20.8–25.9)	23.9 (21.2–26.7)	0.009
Age (yr)	64.9 ± 14.8	61.3 ± 13.8	< 0.001
Sex (Female, %)	250 (41.6)	189 (34.8)	0.02
DM (*n*, %)	247 (41.2)	152 (28.0)	< 0.001
HTN (*n*, %)	377 (62.7)	224 (41.3)	< 0.001
MI (*n*, %)	82 (13.6)	30 (5.5)	< 0.001
CHF (*n*, %)	136 (22.6)	50 (9.2)	< 0.001
CVA (*n*, %)	75 (12.4)	43 (8.0)	0.04
PVD (*n*, %)	32 (5.3)	14 (2.6)	0.02
COPD (*n*, %)	49 (8.2)	31 (5.7)	0.13
Cancer (*n*, %)			
Solid tumor	99 (16.6)	160 (29.5)	
Metastasis	28 (4.7)	40 (7.4)	< 0.001
Leukemia	15 (2.5)	36 (6.6)	
Lymphoma	65 (10.9)	43 (7.9)	
CCI score	3.0 ± 2.3	3.4 ± 2.2	0.002
Sepsis (n, %)	439 (73.0)	358 (65.9)	0.01
Postop AKI (n, %)	23 (3.8)	28 (5.2)	0.32
Cause of CRRT			
Volume overload	94 (15.6)	50 (9.3)	
Metabolic acidosis	126 (21.0)	118 (21.7)	
Hyperkalemia	35 (5.8)	17 (3.1)	0.05
Uremia	50 (8.4)	80 (14.7)	
Oliguria	165 (27.4)	118 (21.7)	
Others	131 (21.9)	160 (29.5)	
CRRT dose (mL/kg)	39.6 ± 12.9	45.1 ± 18.3	< 0.001
MAP (mmHg)	78.4 ± 15.0	76.4 ± 14.1	0.03
Creatinine (mg/dL)	2.6 ± 1.6	2.9 ± 1.6	0.001
eGFR (mL/min/1.73m^2^)	33.6 ± 23.7	29.4 ± 18.4	0.001
Albumin (g/dL)	2.6 ± 0.6	2.6 ± 0.6	0.23
Total cholesterol (mg/dL)	111.9 ± 53.3	98.7 ± 61.4	0.002
WBC (×10^3^/mm^3^)	14.7 ± 14.0	13.4 ± 12.1	0.08
Hemoglobin (g/dL)	9.7 ± 2.4	9.5 ± 2.2	0.04
CRP (mg/L)^a^	64.8 (13.6–156.6)	75.2 (17.5–170.9)	0.12
Vasopressor (*n*, %)	380 (63.3)	419 (77.1)	< 0.001
Ventilator (*n*, %)	418 (77.1)	366 (93.8)	< 0.001
SOFA	9.4 ± 2.3	15.1 ± 1.9	< 0.001
APACHE II	24.6 ± 8.8	29.7 ± 7.1	< 0.001

*Abbreviations*: *DM* diabetes mellitus, *HTN* hypertension, *MI* myocardial infarction, *CHF* congestive heart failure, *CVA* cerebrovascular attack, *PVD* peripheral vascular disease, *COPD* chronic obstructive pulmonary disease, *CCI* Charlson comorbidity index, *AKI* acute kidney injury, *BMI* body mass index, *MAP* mean arterial pressure, *eGFR* estimated glomerular filtration rate, *WBC* white blood cell, *SOFA* sepsis-related organ failure assessment

^a^Data were expressed as a median and interquartile range and compared by Mann Whitney U test

### ICU and hospital stay durations and mortality rates according to BMI classification

Table 3 shows the ICU and hospital stay durations, survival days, and 30-day mortality rates. The mean ICU and hospital stay was 8 (3–17.5) and 20 (6–43) days, respectively. Among patients with high severity, the length of hospital stay was longer in the higher BMI groups than in the underweight group, whereas both ICU and hospital stay were longer in the normal BMI group than others, among those with low severity. During 30 days after CRRT initiation, a total of 701 deaths (61.3%) occurred. The mortality in the obese group was 56.8%, which was significantly lower than in the underweight group (70.7%, *P* = 0.01). Among those with high disease severity, obese group had a significantly lower 30th day mortality than the normal (*P* <  0.001) and underweight group (*P* = 0.007). However, this trend was attenuated in patients with low disease severity (*P* = 0.06).

**Table 3:** Length of stay, survival, and mortality according to BMI classification and disease severity

Disease severity	Variables		BMI classification			Total	*p*-value
		Underweight (*N* = 99)	Normal (*N* = 403)	Overweight (*N* = 220)	Obesity (*N* = 422)		
Low	ICU LOS (d)^a^	5 (2–14)	12 (5–26)	6.5 (3–14.5)	8 (3–15)	8 (3–18)	0.001
	Hospital LOS (d)^a^	9.5 (2.3–44.5)	28 (14–63.5)	25.5 (10–48)	22 (9–42)	23 (10–48)	0.002
	Mortality 30th day (n, %)	38 (61.3)	93 (43.5)	66 (53.2)	97 (48.5)	294 (49.0)	0.06
High	ICU LOS (d)^a^	4 (1–12)	5 (2–14)	5 (3–18)	8 (3–18)	7 (2.5–17)	0.61
	Hospital LOS (d)^a^	5 (2–18.5)	10 (2–27)	16 (3–37)	18.5 (6–40.8)	15 (4–35)	0.002
	Mortality 30th day (n, %)	32 (86.5)	163 (86.2)	70 (72.9)	142 (64.3)	407 (74.9)	< 0.001
Total	ICU LOS (d)^a^	5 (2–14)	9 (3–20)	6 (3–15)	8 (3–16)	8 (3–17.5)	0.03
	Hospital LOS (d)^a^	8 (2–30)	20 (7–46)	23 (6.5–45.5)	21 (8–41)	20 (6–43)	0.006
	Mortality 30th day (n, %)	70 (70.7)	256 (63.5)	136 (61.8)	239 (56.8)	701 (61.3)	0.04

*Abbreviations*: *ICU* intensive care unit, *LOS* length of stay

^a^Data are expressed as a median and interquartile range and compared by Kruskal-Wallis test

### Relationship between BMI and mortality by disease severity in multivariable-adjusted models

**Fig. 2:**
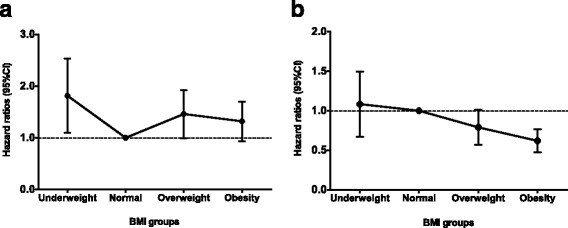
The relationship between BMI groups and 30-day mortality in fully adjusted Cox regression model. **a** low severity, **b** high severity

The association between BMI and mortality was further analyzed using multivariable-adjusted Cox models. To this end, we constructed four different models (Table 4). Overall, obesity was significantly associated with a decreased risk of 30-day mortality in the fully adjusted model (HR, 0.81; 95% CI, 0.68–0.97; *P* = 0.03). In addition, survival benefit of high BMI was also observed when BMI was treated as a continuous variable (HR, 0.97 per 1 kg/m^2^ increase; 95% CI, 0.96–0.99; *P* <  0.001). Notably, the underweight group was associated with an increased risk of death, but it did not reach statistical significance. As mentioned earlier, the relationship between BMI and mortality is confounded by comorbidity conditions. Therefore, we checked an interaction between BMI and disease severity using Cox regression analysis. When the interaction term was included, we found there was a significant interaction between BMI and SOFA score (*P* <  0.001), suggesting the relationship between BMI and mortality was affected by disease severity. Thus, we further analyzed this association in depth according to disease severity (Table 4 and Fig. 2). Among patients with low severity, the underweight patients were at higher risk of 30-day mortality (HR, 1.74; 95% CI, 1.16–2.60; *P* = 0.007) than the normal group. In addition, the overweight and the obese groups also had an increased risk of death as compared with the normal group, resulting in U-shaped risk pattern, although it did not reach the statistical significance in the obese group. In contrast, among those with high severity, obesity was significantly associated with a decreased risk of 30-day mortality (HR, 0.61; 95% CI, 0.48–0.76; *P* <  0.001) as compared with the normal BMI. However, risk of death did not increase in the underweight and the overweight groups. When BMI was analyzed as a continuous variable, high BMI was independently associated with a decreased risk of death only in the high severity group (HR, 0.96 per 1 kg/m^2^ increase; 95% CI, 0.94–0.98; *P* <  0.001), but not in the low severity group.

**Table 4:** Multivariable Cox regression analyses for 30-day mortality

Disease severity	BMI classification	Model 1		Model 2		Model 3		Model 4	
		HR (95% CI)	*p*-value	HR (95% CI)	*p*-value	HR (95% CI)	*p*-value	HR (95% CI)	*p*-value
Low	Underweight	1.57 (1.07–2.30)	0.02	1.66 (1.13–2.45)	0.01	1.74 (1.16–2.60)	0.007	0.99 (0.96–1.01)	0.36
	Normal	1.00 (Reference)		1.00 (Reference)		1.00 (Reference)			
	Overweight	1.44 (1.05–1.99)	0.03	1.48 (1.07–2.04)	0.02	1.41 (1.02–1.94)	0.04		
	Obesity	1.31 (0.98–1.76)	0.07	1.35 (1.01–1.82)	0.04	1.28 (0.95–1.72)	0.11		
High	Underweight	1.07 (0.73–1.56)	0.74	1.03 (0.70–1.51)	0.9	1.04 (0.70–1.53)	0.86	0.96 (0.94–0.98)	< 0.001
	Normal	1.00 (Reference)		1.00 (Reference)		1.00 (Reference)			
	Overweight	0.79 (0.60–1.05)	0.11	0.81 (0.61–1.07)	0.14	0.78 (0.58–1.03)	0.08		
	Obesity	0.63 (0.50–0.79)	< 0.001	0.64 (0.51–0.80)	< 0.001	0.61 (0.48–0.76)	< 0.001		
Total	Underweight	1.27 (0.97–1.66)	0.08	1.27 (0.97–1.67)	0.08	1.28 (0.97–1.68)	0.08	0.97 (0.96–0.99)	< 0.001
	Normal	1.00 (Reference)		1.00 (Reference)		1.00 (Reference)			
	Overweight	1.02 (0.83–1.26)	0.87	1.02 (0.83–1.27)	0.83	0.99 (0.80–1.22)	0.89		
	Obesity	0.84 (0.70–1.01)	0.06	0.86 (0.72–1.03)	0.1	0.81 (0.68–0.97)	0.03		

Model 1: age, sex, CCI score, septic AKI, MAP, eGFR, and SOFA score

Model 2: Model 1 + WBC and albumin

Model 3: Model 2 + CRRT prescription (total effluent volume)

Model 4: Model 3 + BMI as a continuous variable

### Sensitivity analyses

To confirm our findings, we further analyzed in four groups categorized according to BMI of < 18.50 (underweight), 18.50–24.99 (normal), 25.00–27.49 (overweight), and ≥27.5 (obesity) kg/m^2^. In agreement with the findings in Table 4, the association between BMI and mortality by disease severity remained the same after full adjustment of confounders (Additional file 1: Table S1). Furthermore, when we re-defined higher disease severity as APACHE II score of ≥28, survival benefit of obesity was observed only in the high severity group. (Additional file 2: Table S2). This finding was more evidenced by cubic spline curves. The results showed that disease severity assessed by SOFA score was significantly associated with mortality only in non-obese patients, whereas this association was lost in obese patients (Additional file 3: Figure S1).

### Subgroup analyses on the relationship between BMI and mortality according to disease severity

**Fig. 3:**
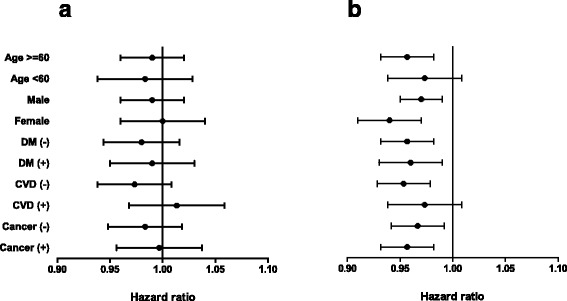
The hazard ratio plot of BMI for 30-day mortality in each subgroup by fully adjusted Cox regression model. BMI was analyzed as a continuous variable. **a** low severity, **b** high severity. *Abbreviations*: DM, diabetes mellitus; CVD, cardiovascular disease; BMI, body mass index

We further analyzed the impact of disease severity on the association between BMI and mortality in several subgroups (Fig. 3), which were stratified by age, sex, diabetes, cardiovascular disease, and cancer. In Cox regression models after full adjustment and BMI as a continuous variable, trends toward a decreased risk of death in high BMI were consistently observed across the subgroups.

## Discussion

In this study, we showed the different relationships between BMI and mortality according to the disease severity in AKI patients undergoing CRRT. Using different multivariable-adjusted models, we found a U-shaped risk pattern of mortality in the low severity group, whereas survival advantage of high BMI was consistently observed in the high severity group. Thus, our findings suggest that disease severity can modify the recently prevailing concept of the “obesity paradox” in critically ill patients undergoing continuous renal replacement therapy.

Previous meta-analyses and observational studies involving a large number of ICU patients have suggested that patients with a higher BMI are more likely to survive than those with a lower BMI [13, 16, 17]. We particularly paid attention to patients with AKI requiring CRRT. Critically ill patients are generally hypercatabolic and have substantial energy expenditure in proportion to high level of stress [25, 26]. In addition, patients with AKI have a high prevalence of malnutrition [27], and protein is excessively degraded by AKI-induced uraemia [28]. Notably, loss of protein is accelerated in AKI patients undergoing CRRT because protein and other nutritional elements are lost through CRRT [29]. It was also found that centrally infused protein losses into CRRT effluent range from 10 to 17% [30–32]. In this regard, AKI patients on CRRT are more susceptible to a loss of energy reserve. The unique characteristics of these patients can explain why high BMI provided survival advantage in patients with high disease severity.

Conversely, high BMI itself has been considered as a significant risk factor of AKI. A recent observational cohort studies found that obese patients were at a higher risk of developing AKI than patients with normal BMI [6, 7]. Interestingly, BMI plays a different role in acutely ill patients after having AKI. In a study by Druml et al. [7] involving 5232 patients with ‘failure’ stage by RIFLE criteria, patients with BMI of 30–35 kg/m^2^ had the lowest risk of death compared to those with other BMI categories. This finding was contradicted by Danziger et al. [6]. They found that relative risks of hospital and l-year mortality in obese AKI group were greater than other BMI groups. This discrepancy can partially be explained by different disease severity between the studies. In fact, in the study by Druml et al., the median value of the Simplified Acute Physiology Score II was 47 and overall ICU and hospital mortality rates were 56.1% and 63.9%. However, disease severity was much lower in the study by Danziger et al., where overall hospital mortality was only 10% and baseline serum creatinine was 1.1–1.4 mg/dL without detailed information of SOFA score. It is possible that there is a biphasic role of obesity depending on disease severity. To corroborate this, we found that high BMI was consistently associated with a decreased risk of death among patients with high severity, but there was a U-shaped pattern for mortality among those with low severity.

It is generally accepted that fat tissue can function as an energy reservoir. In addition, severe illness can worsen high catabolic state, protein loss, and muscle wasting. Thus, when disease is severe, obese patients having high energy store can tolerate stressful and damaging conditions better than non-obese patients [33]. In contrast, energy-storage role of fat do not appear to play a significant role in patients with low disease severity. Presumably, energy-consuming process and muscle wasting are diminished when disease burden is less severe. The relatively well-preserved nutritional status in patients with low disease severity can also support the findings of favourable effect between obesity and mortality in this group. A recent study by Robinson et al. investigated the relationship among obesity, nutritional status, and mortality [19]. They showed that high BMI was significantly associated with survival benefit in critical ill patients. Of note, in their findings, malnutrition was less prevalent in obese patients than in underweight and normal patients, suggesting nutrition as a potential factor to explain survival advantage of obesity. Thus, when nutritional status is poor accompanied by high disease severity, energy-storing fat can compensate for the loss of energy and decline in nutritional status. Conversely, better nutritional status in low disease severity can attenuate the favourable effects of high BMI. In this regard, future studies should address the association between BMI, disease severity, and nutritional status in critically ill patients with AKI.

There are several limitations that should be discussed in this study. First, to clarify the association between BMI and mortality, we constructed various multivariable models adjusted for many potential factors. However, this is an observational study and unknown bias may affect the study results and our findings need to be interpreted with caution. Second, only BMI was applied to define obesity. BMI provides an easy way to measure obesity and has been widely used. However, BMI has been criticized because it is not an accurate measure of fat [34]. Other parameters such as waist circumference or abdominal diameter can be added to increase the diagnostic accuracy for obesity [35]. Unfortunately, such measurement was not easily feasible in the ICU setting, particularly when patients are in critical condition. Third, we used the BMI classification proposed by the Committee of Clinical Practice Guidelines and Korean Society for the Study of Obesity [23]. When the WHO international criteria were applied, only 6.9% of patients had BMI > 30 kg/m^2^ in our study. Thus, analysis for morbid obese patients was not feasible and result of our study may not be applicable to other ethnic groups, whose BMI is higher than our population. We confirmed our findings using a different flexible BMI classification for the Asian population proposed by the WHO [24] as indicated in Additional file 1: Table S1. Although we defined obesity as BMI ≥ 27.5 kg/m^2^, the results were unaltered. Nevertheless, we acknowledge well that BMI cut-off point for obesity in this study is lower than that in other Western countries and thus our findings may not be extrapolated to such extremely obese patients. Further studies with large number of obese patients should focus on this issue. Fourth, our database did not have much information on nutritional indices, thus nutritional status could not be thoroughly evaluated. However, all nutritional supports including whether or not to start enteral or parenteral nutrition were precisely decided by dietitians and intensivists upon ICU admission in our centers. Finally, CRRT prescription was different across BMI and disease severity groups. Patients with high BMI tended to have less amount of dialysis, and this tendency was persistent in both disease severity groups (data not shown). We showed better survival of high BMI patients even with low dose of dialysis; however, it is uncertain whether CRRT dose can affect to mortality in AKI patients undergoing CRRT [36].

## Conclusions

In conclusion, this study showed that high BMI is associated with survival benefit in AKI patients undergoing CRRT. However, this association was observed only in patients with high disease severity. Our findings suggest that disease severity can modify an inverse relationship between high BMI and mortality in these patients. Thus, interpretation of this relationship should be made with caution depending on disease severity. Further studies are required to evaluate whether BMI can be incorporated into risk stratification depending on disease severity in critically ill patients.

## Additional files

Additional file 1: Table S1. Multivariate Cox regression analyses for 30-day mortality by WHO classification. (DOCX 18 kb)

Additional file 2: Table S2. Multivariate Cox regression analyses for 30-day mortality by APACHE II score. (DOCX 18 kb)

Additional file 3: Figure S1. The cubic spline curves for 30-day mortality according to SOFA score. (a) non-obese group, (b) obese group. *Abbreviations*: SOFA, sepsis-related organ failure assessment. (PDF 737 kb)

## Abbreviations

AKI: Acute kidney injury
APACHE: Acute Physiology and Chronic Health Evaluation
BMI: Body mass index
CCI: Charlson comorbidity index score
CI: Confidence interval
CKD: Chronic kidney disease
CRP: C-reactive protein
CRRT: Continuous renal replacement therapy
DM: Diabetes mellitus
eGFR: estimated glomerular filtration rate
ESRD: End-stage renal disease
HR: Hazard ratio
ICU: Intensive care unit
IRB: Institutional review board
SOFA: Sepsis-related organ failure assessment
WBC: White blood cell
WHO: World Health Organization
