# Supplementary material for: The impact of disease severity on paradoxical association between body mass index and mortality in patients with acute kidney injury undergoing continuous renal replacement therapy
Source: BMC Nephrol. 2018 Feb 7;19:32. doi: 10.1186/s12882-018-0833-5 (PMC5804063; doi:10.1186/s12882-018-0833-5)
Supplement: Supplementary file 1 — Multivariate Cox regression analyses for 30-day mortality by WHO classification. (DOCX 18 kb) [file 12882_2018_833_MOESM1_ESM.docx]

**Table S1. Multivariate Cox regression analyses for 30-day mortality by WHO classification**

| **Disease**  **severity** | **BMI classification** | **Model 1** | | **Model 2** | | **Model 3** | | **Model 4** | |
| --- | --- | --- | --- | --- | --- | --- | --- | --- | --- |
|  |  | **HR (95% CI)** | **p-value** | **HR (95% CI)** | **p-value** | **HR (95% CI)** | **p-value** | **HR (95% CI)** | **p-value** |
| **Low** | **Underweight** | 1.39 (0.98-1.98) | 0.07 | 1.45 (1.01-2.08) | 0.04 | 1.54 (1.06-2.24) | 0.02 | 0.99 (0.96-1.01) | 0.36 |
|  | **Normal** | 1.00 (Reference) |  | 1.00 (Reference) |  | 1.00 (Reference) |  |  |  |
|  | **Overweight** | 1.29 (0.94-1.79) | 0.12 | 1.32 (0.95-1.83) | 0.1 | 1.29 (0.93-1.78) | 0.13 |  |  |
|  | **Obesity** | 1.01 (0.72-1.42) | 0.94 | 1.03 (0.73-1.45) | 0.86 | 0.97 (0.69-1.37) | 0.87 |  |  |
| **High** | **Underweight** | 1.05 (0.72-1.52) | 0.81 | 1.02 (0.70-1.48) | 0.94 | 1.04 (0.71-1.52) | 0.84 | 0.96 (0.94-0.98) | <0.001 |
|  | **Normal** | 1.00 (Reference) |  | 1.00 (Reference) |  | 1.00 (Reference) |  |  |  |
|  | **Overweight** | 0.74 (0.56-0.96) | 0.02 | 0.72 (0.55-0.94) | 0.02 | 0.70 (0.54-0.92) | 0.01 |  |  |
|  | **Obesity** | 0.62 (0.47-0.82) | 0.001 | 0.95 (0.49-0.85) | 0.002 | 0.62 (0.47-0.82) | 0.001 |  |  |
| **Total** | **Underweight** | 1.21 (0.94-1.56) | 0.14 | 1.22 (0.94-1.58) | 0.13 | 1.24 (0.95-1.61) | 0.11 | 0.97 (0.96-0.99) | <0.001 |
|  | **Normal** | 1.00 (Reference) |  | 1.00 (Reference) |  | 1.00 (Reference) |  |  |  |
|  | **Overweight** | 0.94 (0.77-1.15) | 0.55 | 0.94 (0.76-1.15) | 0.52 | 0.91 (0.74-1.11) | 0.35 |  |  |
|  | **Obesity** | 0.74 (0.60-0.91) | 0.005 | 0.77 (0.62-0.95) | 0.02 | 0.73 (0.59-0.91) | 0.004 |  |  |

Model 1: age, sex, CCI score, septic AKI, MAP, eGFR, and SOFA score

Model 2: Model 1 + WBC and albumin

Model 3: Model 2 + CRRT prescription (total effluent volume)

Model 4: Model 3 + BMI as a continuous variable
