# Supplementary material for: The impact of disease severity on paradoxical association between body mass index and mortality in patients with acute kidney injury undergoing continuous renal replacement therapy
Source: BMC Nephrol. 2018 Feb 7;19:32. doi: 10.1186/s12882-018-0833-5 (PMC5804063; doi:10.1186/s12882-018-0833-5)
Supplement: Supplementary file 2 — Multivariate Cox regression analyses for 30-day mortality by APACHE II score. (DOCX 18 kb) [file 12882_2018_833_MOESM2_ESM.docx]

**Table S2. Multivariate Cox regression analyses for 30-day mortality by APACHE II score**

| **Disease**  **severity** | **BMI classification** | **Model 1** | | **Model 2** | | **Model 3** | | **Model 4** | |
| --- | --- | --- | --- | --- | --- | --- | --- | --- | --- |
|  |  | **HR (95% CI)** | **p-value** | **HR (95% CI)** | **p-value** | **HR (95% CI)** | **p-value** | **HR (95% CI)** | **p-value** |
| **Low** | **Underweight** | 1.31 (0.88-1.95) | 0.19 | 1.38 (0.92-2.08) | 0.12 | 1.37 (0.91-2.09) | 0.14 | 0.99 (0.96-1.02 | 0.41 |
|  | **Normal** | 1.00 (Reference) |  | 1.00 (Reference) |  | 1.00 (Reference) |  |  |  |
|  | **Overweight** | 1.25 (0.91-1.72) | 0.17 | 1.20 (0.87-1.66) | 0.26 | 1.18 (0.86-1.63) | 0.31 |  |  |
|  | **Obesity** | 1.03 (0.75-1.41) | 0.87 | 1.05 (0.76-1.45) | 0.77 | 0.99 (0.72-1.38) | 0.99 |  |  |
| **High** | **Underweight** | 1.13 (0.81-1.58) | 0.48 | 1.06 (0.75-1.50) | 0.73 | 1.05 (0.74-1.48) | 0.8 | 0.98 (0.96-0.99) | 0.04 |
|  | **Normal** | 1.00 (Reference) |  | 1.00 (Reference) |  | 1.00 (Reference) |  |  |  |
|  | **Overweight** | 1.00 (0.76-1.31) | 0.99 | 1.01 (0.77-1.32) | 0.96 | 1.00 (0.76-1.32) | 0.99 |  |  |
|  | **Obesity** | 0.67 (0.50-0.89) | 0.006 | 0.70 (0.52-0.94) | 0.02 | 0.70 (0.52-0.93) | 0.02 |  |  |
| **Total** | **Underweight** | 1.19 (0.92-1.54) | 0.18 | 1.18 (0.91-1.53) | 0.21 | 1.17 (0.90-1.53) | 0.24 | 0.98 (0.97-0.99) | 0.04 |
|  | **Normal** | 1.00 (Reference) |  | 1.00 (Reference) |  | 1.00 (Reference) |  |  |  |
|  | **Overweight** | 1.10 (0.90-1.35) | 0.34 | 1.09 (0.88-1.34) | 0.43 | 1.08 (0.87-1.32) | 0.49 |  |  |
|  | **Obesity** | 0.81 (0.66-1.01) | 0.06 | 0.84 (0.68-1.05) | 0.12 | 0.83 (0.67-1.02) | 0.08 |  |  |

Model 1: age, sex, CCI score, septic AKI, MAP, eGFR, and APACHE II score

Model 2: Model 1 + WBC and albumin

Model 3: Model 2 + CRRT prescription (total effluent volume)

Model 4: Model 3 + BMI as a continuous variable
