# Supplementary material for: The impact of disease severity on paradoxical association between body mass index and mortality in patients with acute kidney injury undergoing continuous renal replacement therapy
Source: BMC Nephrol. 2018 Feb 7;19:32. doi: 10.1186/s12882-018-0833-5 (PMC5804063; doi:10.1186/s12882-018-0833-5)
Supplement: Supplementary file 3 — The cubic spline curves for 30-day mortality according to SOFA score. (a) non-obese group, (b) obese group. Abbreviations: SOFA, sepsis-related organ failure assessment. (PDF 737 kb) [file 12882_2018_833_MOESM3_ESM.pdf]

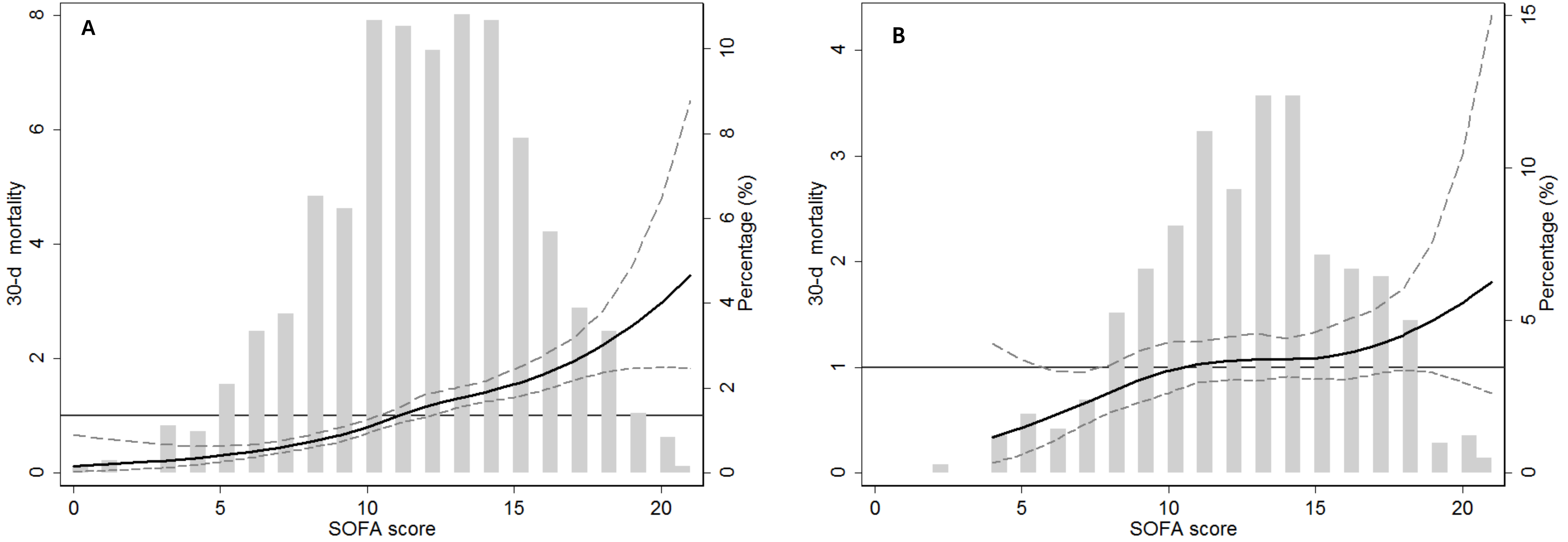

Figure S1. The cubic spline curves for 30-day mortality according to SOFA score. (a) non-obese group, (b) obese group, Abbreviations: SOFA, sepsis-related organ failure assessment
